# Integration of automation into an existing clinical workflow to improve efficiency and reduce errors in the manual treatment planning process for total body irradiation (TBI)

**DOI:** 10.1002/acm2.12894

**Published:** 2020-05-19

**Authors:** David H. Thomas, Brian Miller, Rachel Rabinovitch, Sarah Milgrom, Brian Kavanagh, Quentin Diot, Moyed Miften, Leah K. Schubert

**Affiliations:** ^1^ Department of Radiation Oncology University of Colorado Aurora CO USA

**Keywords:** automation, incident learning systems, TBI

## Abstract

**Purpose:**

To identify causes of error, and present the concept of an automated technique that improves efficiency and helps to reduce transcription and manual data entry errors in the treatment planning of total body irradiation (TBI).

**Methods:**

Analysis of incidents submitted to incident learning system (ILS) was performed to identify potential avenues for improvement by implementation of automation of the manual treatment planning process for total body irradiation (TBI). Following this analysis, it became obvious that while the individual components of the TBI treatment planning process were well implemented, the manual ‘bridging’ of the components (transcribing data, manual data entry etc.) were leading to high potential for error. A C#‐based plug‐in treatment planning script was developed to remove the manual parts of the treatment planning workflow that were contributing to increased risk.

**Results:**

Here we present an example of the implementation of “Glue” programming, combining treatment planning C# scripts with existing spreadsheet calculation worksheets. Prior to the implementation of automation, 35 incident reports related to the TBI treatment process were submitted to the ILS over a 6‐year period, with an average of 1.4 ± 1.7 reports submitted per quarter. While no incidents reached patients, reports ranged from minor documentation issues to potential for mistreatment if not caught before delivery. Since the implementation of automated treatment planning and documentation, treatment planning time per patient, including documentation, has been reduced; from an average of 45 min pre‐automation to <20 min post‐automation.

**Conclusions:**

Manual treatment planning techniques may be well validated, but they are time‐intensive and have potential for error. Often the barrier to automating these techniques becomes the time required to “re‐code” existing solutions in unfamiliar computer languages. We present the workflow here as a proof of concept that automation may help to improve clinical efficiency and safety for special procedures.

## 1 INTRODUCTION

Delivery of a wide range of radiation therapy (RT) treatments in a modern Radiation Oncology department requires a complex set of processes ranging from simulation, contouring target volumes and normal tissue structures, treatment planning, dose delivery, and quality assurance. These tasks range from repetitive labor‐intensive manual tasks to fully automated computer‐driven procedures, and workflows, equipment, and software are often unique to each clinic. Recent developments in information technology have enabled reducing the requirements of manual procedures in the treatment planning workflow.^1^ These efforts have been made in part due to the priorities set by professional organizations including American Society for Radiation Oncology (ASTRO), American Association of Physicists in Medicine (AAPM), and the American College of Radiology (ACR).^1^, ^2^, ^3^ Several vendors of treatment planning systems (TPS) now offer a Scripting Application Programming Interface (API) to enable clinics to develop their own automated computer‐driven processes that are customized to their clinical workflow.^4^ Various studies have shown that scripting can be used in automated treatment plan optimization, and data‐mining, and it offers advantages in both safety and productivity when repetitive manual data entry tasks can be efficiently automated.^2^, ^3^, ^5^, ^6^, ^7^

While treatment planning processes in the clinic are becoming more complex in general, there remain many manual treatment planning procedures that clinics rely on for simple treatment types or special procedures. While the rate of errors in RT delivery is low,^1^ each step of the manual treatment planning process can introduce potential for catastrophic errors. Manual tasks have higher potential for error, compared with automated tasks,^1^, ^8^ and automation is high on the hierarchy of intervention effectiveness.^9^

One such manual procedure, total body irradiation (TBI), is an example of a simple treatment planning procedure that nonetheless contains many manual data entry tasks, especially when three‐dimensional (3D) imaging is not acquired for dose calculation. Total body irradiation is a high‐dose myeloablative therapy used, for example, prior to allogeneic stem cell transplant. TBI is a potentially fatal therapy, without appropriate medical monitoring. Attention to dose calculations, treatment timing, treatment parameters, and treatment technique are essential to minimize the risk of acute and late morbidity and mortality.^10^ When volumetric imaging is not acquired, a “hand‐calculation” method is typically used to calculate monitor units (MU). In this case the treatment planning process generally involves manually transcribing information from the physician’s prescription into the hand calculation, and then manually entering the subsequent field information into the TPS. TBI treatments are unique compared with conventional treatments in that radiation delivery must occur on specific dates that correspond with the transplant timeline. This requirement can potentially increase urgency and pressure during the planning process, which are factors that can contribute to errors.^11^ There may be instances that necessitate recalculations “on‐the‐fly.” Examples include a treatment machine going down requiring recalculation for the backup machine, or patient weight loss leading to separation changes requiring recalculation of monitor units. It has been shown that TBI leads to a higher proportion of high‐risk events compared to non‐TBI‐related treatments.^11^

**Fig. 1:**
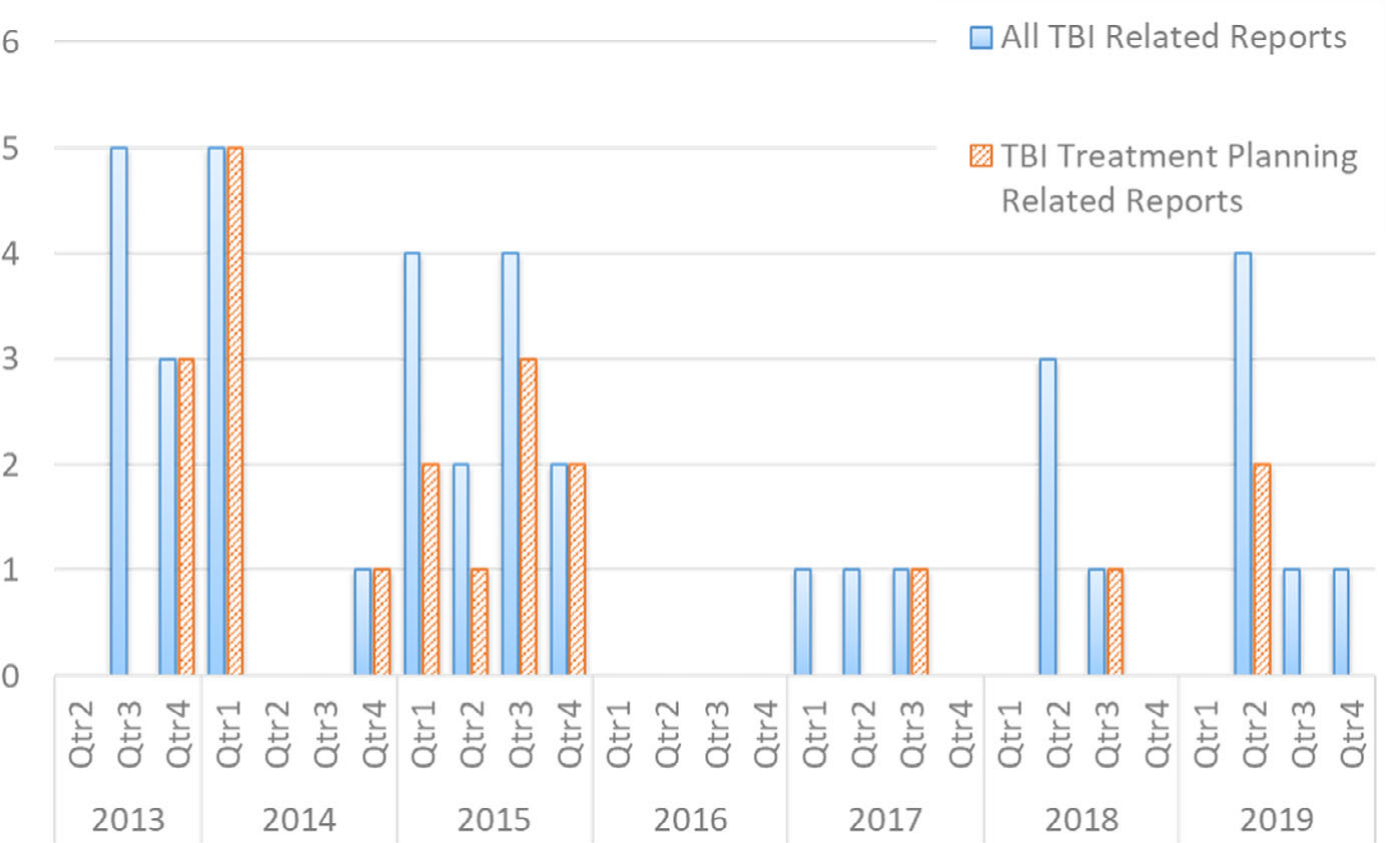
Number of TBI treatment planning‐related reports submitted to the ILS per quarter over a 6‐yr period. An average of 1.4 ± 1.7 reports were submitted per quarter prior to the implementation of automation. Automation was implemented at the end of Quarter 1 of 2019.

Incident learning is a safety improvement tool which involves the reporting and analysis of incidents and near misses. From the analysis of these reports, interventions can be put in place to prevent, better detect, or mitigate similar events in the future, thereby improving patient safety.^11^, ^12^, ^13^, ^14^ Varying types of interventions can be implemented, with varying levels of effectiveness and are typically classified into a hierarchy of effectiveness, in which forcing functions and automation are the most effective.^13^ Recent studies from departments using incident learning suggest, however, that forcing functions and automation are less commonly implemented in practice than less effective interventions.^11^, ^15^ Information submitted to our departmental incident learning system (ILS) over a period of 6 yr was analyzed to identify causes of errors in the TBI planning process. Incident reports were analyzed to identify potential avenues for improvement by implementing a highly effective intervention, specifically automation of the manual treatment planning process. Figure 1 shows a histogram of the number of TBI‐related reports submitted to the ILS per quarter over a 6‐year period. None of these potential errors reached the patient, and they were generally caught either during the physics initial chart check or during the therapists' pretreatment checks.

Here we present an example of the implementation of “Glue” programming, combining Eclipse Scripting Application Programming Interface (ESAPI) C# scripts with existing Excel Calculation Worksheets, to improve clinical efficiency and safety within the treatment planning workflow.“Glue” programming refers to a programming technique of passing data through multiple applications, allowing us to take advantage of existing clinical workflows to fully automate a clinical task that was previously manual.

## 2 MATERIALS AND METHODS

### 2.A Treatment technique

**Fig. 2:**
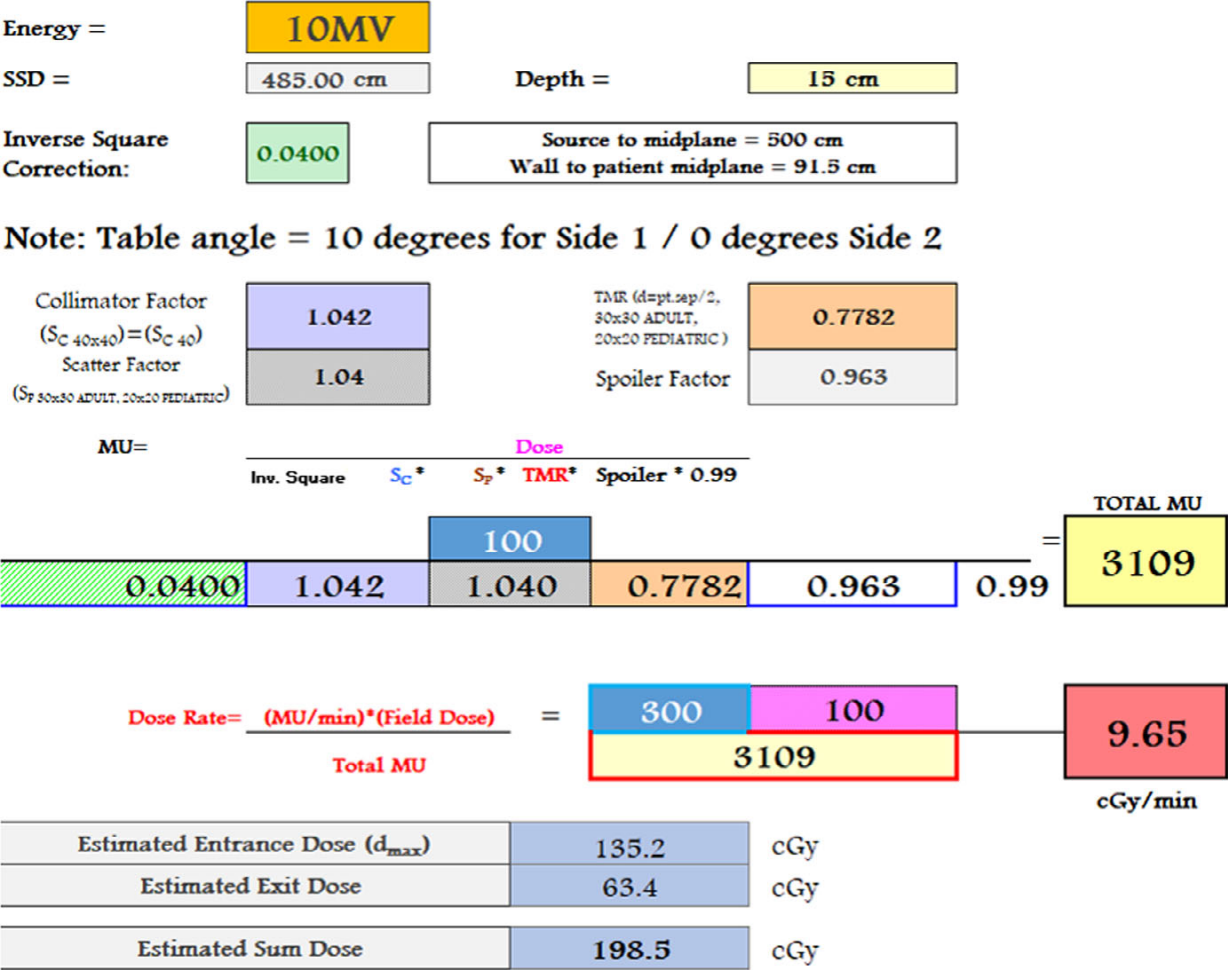
Example of total body irradiation manual monitor unit “hand” calculation excel spreadsheet used to calculate the total MU and dose rate per field. Prior to automation, values were manually entered from look‐up tables. Following automation, C#/ESAPI script interacted with the spreadsheet using the Microsoft Office Component Object Model Interface to edit the input cells, and pass back the required MU and dose rate [MU/min] values to the TPS.

Total body irradiation is performed in our clinic using extended SSD on a Varian TrueBeam (Varian Medical Systems). Typically, no volumetric imaging (CT) is acquired, and MU calculation is not performed in the Treatment Planning system (Eclipse).A whole body midplane dose uniformity of ±10% is achieved using a “hand‐calculation” method to determine the monitor units (MU) and dose rate. Various clinical protocols are used at our institution, depending on the clinical indications and patient's age. Tissue compensators are used to achieve the required uniformity. A beam spoiler is used to increase superficial dose and satisfy homogeneity requirements. Thermoluminescent dosimeters (TLDs) are used to monitor entrance and exit dose on the first fraction of treatment, and additional boost radiation is delivered when required. Depending on protocol, patients are treated with equally weighted AP/PA with the patient in a lateral decubitus position, or with opposed lateral fields with the patient supine. All patients are treated lying down. A custom positioning device is used with a raised gurney in each case. Custom lung shielding blocks are used when required (depending on clinical protocol), and portal films confirming positioning accuracy performed prior to treatment. The instantaneous dose rate at prescription point is typically limited to 10 cGy/min per AAPM’s TG‐29 by clinical protocol, but can be as high as 15 cGy/min depending on clinical protocol^16^, ^17^, ^18^, ^19^, ^20^. Figure 2 shows an example calculation. Absolute dose is calibrated at 100 cm SAD (surface to axis distance) with a 10‐×‐10‐cm field size. TBI treatments are delivered using a 40‐×‐40‐cm field and extended SSD of 500 cm. Thus, the calculation of monitor units (MU) includes a combination (a) *patient‐specific factors* and (b) *machine/technique standard factors*.

- *Patient‐specific factors*, as follows*:* prescription dose [cGy], patient separation [cm], maximum allowed dose rate [cGy/min].
- *machine/technique standard factors*: inverse square factor, collimator factor (S_c_), scatter factor (S_p_), tissue maximum ratio (TMR), spoiler factor, and 0.99 dose‐to‐water to dose‐to‐muscle correction factor.^21^

These factors are entered into an Excel spreadsheet to calculate the MU per field and required field dose rate [MU/min] to achieve the maximum dose rate [cGy/min] allowed in the prescription. Information from the spreadsheet, and from knowledge of standard beam geometry, is then used to manually create beams in the TPS and manually enter all beam parameters.

### 2.B Incident analysis

Our departmental ILS was implemented in 2013.^15^ The main design goal of the ILS was to create a highly sensitive system to capture as much information throughout the department as possible. Reports were classified according to incidents and near misses involving therapeutic radiation, imaging/simulation, and patient care (not involving radiation), unsafe conditions, operational issues, and accolades/suggestions. Treatment type/technique is one of the classifiers of each report. Reports were analyzed to identify causes and contributing factors.

### 2.C Implementation of automation

This work was performed on Varian Eclipse treatment planning system v15.6 using ESAPI, a C# programming‐based toolbox, highly integrated with the Eclipse treatment planning system.

**Fig. 3:**
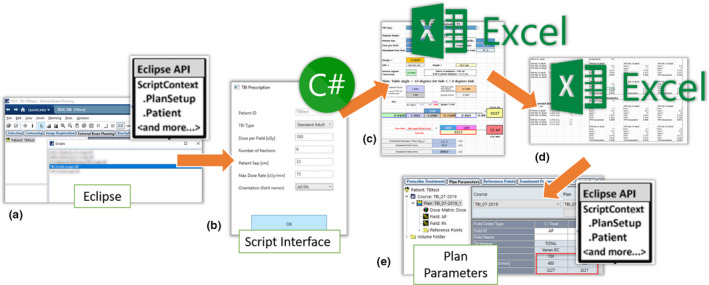
Workflow of the total body irradiation autoplanning script, which requires only a single user interaction to automatically generate a TBI plan. (a) A patient is opened and a plug‐in ESAPI script is run in Eclipse. (b) ESAPI sends patient information to a custom GUI interface written in C#. Prescription details are entered manually by the planner into the GUI. (c) C# script uses Microsoft Office component object model interface to open and interact with an Excel file, transposing the patient information and prescription details. (d) Dose rate and MU per field are calculated from previously validated interpolated TMR look‐up tables in the Excel file. (e) ESAPI is used to create a plan and field parameters are automatically entered into the plan using the data from Excel file. The Excel file is uploaded to the Record and Verify System for physics check prior to treatment, and the plan is ready for plan approval.

C#‐based scripts were developed in ESAPI to interact with existing Excel spreadsheets to calculate the field parameters (MU and dose rate) for a TBI treatment plan, and operated as follows;

- ***ESAPI:*** A selected patient is opened in ARIA, and the ESAPI plug in script is called from External Beam Planning (“Eclipse”) [Fig. 3(a)]. The plug‐in script has a custom graphical user interface (“GUI”) which allows treatment parameters to be entered [Fig. 3(b)]. Specifically, the parameters to be entered include TBI Type (“standard adult”, “standard pediatric” etc.), dose per field [cGy], number of fractions, patient separation [cm], maximum dose rate [cGy/min], and patient orientation for treatment (field names, e.g., “AP/PA”, “RLat/LLat”).
- ***C#:*** Using the Microsoft Office component object model interface, the GUI passes these data, along with patient name and MRN, to an existing password protected “master” Excel spreadsheet located on the ARIA server, which is copied and saved to a temporary folder [Fig. 3(c)]. The Object Model Interface edits the appropriate cells in the Excel file to transpose the patient information and prescription data.
- ***Excel:*** To calculate the field parameters, TBI Factors and TMR tables are contained in the Excel spreadsheet (Fig. 2). The patient separation is used to choose the required beam energy from a look up table, and machine‐specific factor interpolation is performed in Excel [Fig. 3(d)] to calculate total MU and required field dose rate [MU/min] to achieve the maximum dose rate [cGy/min] allowed in the prescription, using the equation shown in Fig. 2. The patient‐specific Excel file is then automatically printed to PDF format and uploaded to the Record and Verify system. Using the Object Model Interface, the field parameters are passed back from the excel file to the ESAPI interface by the C# code.
- ***ESAPI:*** ESAPI is then used to automatically generate a plan in Eclipse, performing the following steps without any user interaction [Fig. 3(e)]. Modification of the Eclipse/ARIA database in this way is only possible in Eclipse v15 onward, and should be done only with extreme care, as there exists the very obvious potential for error. For example errors may include, but are not limited to, accidental plan deletion or patient corruption requiring the patient to be purged from the database resulting in data loss. ESAPI scripts were created in a test environment (“test‐box”) and rigorously validated before being copied to the clinical environment for clinical use. The ESAPI script performed the following fully automated steps:
  1. A 40 × 40 × 40 cm water phantom and structure set containing a “body” contour is added to the current patient.
  2. A new course and plan are inserted, with the Course and Plan name based on the date the script was run. Numbers are appended to the names if the plan already exists, following the standard Eclipse naming conventions (“TBI_07‐2019‐1”, “TBI_07‐2019‐2”, etc.)
  3. Appropriately named reference points are inserted and reference point dose limits are set based on the prescription information.
  4. Static fields are added with appropriate isocenter and field sizes set, and the TBI‐specific technique “TOTAL” applied to the fields.
  5. In order to be able to set the MUs per field, a dose distribution must be present in the plan. Therefore, a low‐resolution dose calculation is performed using Analytical Anisotropic Algorithm (AAA). This dose distribution is irrelevant for treatment. The per‐field MUs are set by adjusting a combination of the plan normalization factor and the field weight factor in order to normalize each field appropriately to achieve the MUs calculated in the Excel file.
  6. The patient data are saved and the script exits notifying the user that a plan has been created.
  7. The Excel file is opened for the user to view, and verify that the treatment parameters have been set correctly. The plan is then ready for Plan Approval and scheduling.

### 2.D Quality assurance

As automation takes a larger role in RT treatment planning, a robust clinical quality assurance system is required that tests and maintains the automation process.^22^ Dosimetric quality of the automated TBI planning was assessed here by verifying that the system is equivalent to existing manual processes by replanning of previous TBI patients to ensure plan quality. As opposed to automated inverse treatment planning implementations^23^, ^24^, ^25^, ^26^ which require rigorous dosimetric validation to ensure that quality of the treatment plan is not compromised, the output of the automated planning system implemented for TBI is exactly equivalent to a manually generated plan. The treatment plans for 25 patients who received TBI previously, based on hand calculations, were recalculated using the automated planning script to ensure equivalence in all relevant plan parameters, and a subset of these plans are replanned periodically.

## 3 RESULTS

Over a 6.5‐yr period (from January 2013 to June 2019), a total of 9108 distinct patients were treated in our department. A total of 411 distinct TBI patients were treated, which accounted for 4.5% of distinct patients treated. Over this same period, a total of 1566 reports were submitted to the ILS (Fig. 1), the majority of which (80%) impacted technical aspects of treatment (simulation, planning, and treatment delivery). About 470 reports (30% of the total) were treatment planning related. Thirty‐five reports were related to TBI treatments (average of 1.4 ± 1.7 reports submitted per quarter), of which 24 were treatment planning related. These reports ranged from minor documentation errors to dose calculation errors with the potential for mistreatment if not caught before delivery. Of the reports concerning TBI, 1% (n = 11) were related to treatment, 34% (n = 12) to documentation, and 34% (n = 12) to treatment field issues (miscalculation, transcription errors, etc.). In total, TBI accounted for 5.1% of all treatment planning‐related reports, so TBI was slightly overrepresented compared to the proportion of total treatment planning reports by a factor of x1.1. None of these potential errors reached the patient. They were generally caught either during the physics initial chart check or during the therapists’ pretreatment checks. About 49% (n = 17) of reports were classified as operational/workflow issues, 31% (n = 11) were classified as unsafe condition, and 20% (n = 7) were classified as near‐miss. Table 1 shows examples of the most common issues identified.

**Table 1:** Examples of errors related to TBI planning submitted to the departmental incident learning system (ILS).

TBI planning error type	Root errors detected	Cause(s)	Significance	Detected by
Documentation error	Incorrect patient name in plan document in R&V	Manual data entry error	Minor significance; potential to cause treatment delay	Physics check
Documentation error	Field names mislabeled (all fields labelled “LLat”)	Manual data entry error	Minor significance; potential to cause treatment delay	Physics check
Documentation error (incomplete)	Missing calculation document in R&V	Manual documentation step missed in the treatment planning process	Minor significance; potential to cause treatment delay	Therapist check
Documentation error	Incorrect plan type used for calculation (adult patient, calculated on pediatric calculation worksheet)	Manual data entry error	Potential for mistreatment if not caught before delivery	Physics check
Manual field entry	Treatment time for fields set incorrectly	Pediatric patient requiring low dose‐rate, requiring longer treatment time than standard	Minor significance; potential to cause treatment delay	Therapist check
Manual field entry	Gantry angle set incorrectly (set to 270° instead of 90°)	Trainee dosimetrist unfamiliar with the treatment technique.	Unlikely to cause mistreatment; potential to cause treatment delay	Physics check
Manual field entry	Incorrect field size set in TPS	Manual data entry error	Unlikely to cause mistreatment; potential to cause treatment delay	Physics check
Data transcribed incorrectly	Patient separation transcribed from Rx incorrectly	Manual data entry error	Potential for mistreatment if not caught before delivery	Physics check
TPS user error	Field MUs incorrectly set (531 MU instead of 231 MU)	Manual data entry error	Potential for mistreatment if not caught before delivery	Physics check
Data look‐up incorrect	MU calculated using Spoiler Factor for incorrect energy	Manual data entry error.	Potential for mistreatment if not caught before delivery	Physics check
TPS user error	TBI dose rate incorrect following a replan for back‐up machine	Plan was manually copied, dose‐rate not changed	Potential for mistreatment if not caught before delivery	Therapist check
TPS user error	TBI dose rate incorrect for one of two fields	Manual data entry error	Potential for mistreatment if not caught before delivery	Therapist check
TPS user error	Energy set incorrectly in TPS	Manual data entry error	Potential for mistreatment if not caught before delivery	Physics check

Since the implementation of automated treatment planning and documentation in the first quarter of 2019, treatment planning time per patient, including documentation, has been reduced from an average of 45 min pre‐automation to <20 min post‐automation. This was evaluated using a stopwatch to time a dosimetrist performing the entire planning process (three cases in each case). No treatment planning‐related incidents have been submitted in the two quarters since implementation.

## 4 DISCUSSION

Our departmental ILS includes near‐miss events and other safety‐related reports. These events represent useful learning opportunities, which we have used here to evaluate a manual treatment planning process and improve the workflow with the implementation of automation scripting of the TPS.

Following ILS analysis, it became obvious that although individual components of the TBI treatment planning process were well implemented, the manual “bridging” of the components (transcribing data, manual data entry etc.) was leading to high potential for error. The solution that was implemented is an example of “Glue” programming, combining automated scripts with existing calculation frameworks to improve clinical efficiency and safety within the treatment planning workflow. A small utility that uses the scripting API within Eclipse was created to automate existing manual tasks. Critical parts of the process, for example TMR interpolation for MU calculation, are performed in existing well‐validated spreadsheets. We used Excel worksheets, which are well‐suited for these calculations, because they are password protected and can be updated with new or additional beam configuration data easily. Patient information and field parameters are passed back to ESAPI to generate plans using automation tools available in the latest version of Eclipse (v15.6).

Since the pre‐automation rate of incidents per quarter is low (1.4 ± 1.7 reports per quarter), it is currently premature to draw the conclusions on the risk reduction with only two quarters of data. Scripting and automation of repetitive manual data entry tasks offer advantages in both safety and productivity. In addition to TBI, other types of treatments that are well‐suited to such an intervention include Total Skin Electron (TSE) and clinical electron treatments, because “hand calculations” are preferred by many clinics over more complex treatment planning algorithms (for instance, electron Monte Carlo). While the implementation of automated treatment planning procedures offers obvious advantages to quality and efficiency improvements, clinical implementation is often limited due to the effort and knowledge level required to implement automated scripted versions of existing techniques. Existing techniques, while suboptimal and time intensive, are likely well‐validated, and the barrier to automating them becomes the time required to “re‐code” existing solutions in sometimes unfamiliar computer languages. For example, designing a functional user interface or report which will display well on a range of interfaces that exist in a typical Radiation Oncology department (Citrix clients, Eclipse workstations, hospital PCs etc.) is a nontrivial task, which can be time‐intensive and frustrating for a clinical medical physicist. Often, a simple Excel worksheet has already been created to achieve the task; complex mathematical functions and helpful conditional formatting can be created in minutes using the basic Office interface, or simple Visual Basic for Applications (VBA) programming. However, such use of Excel spreadsheets requires manual data entry and, thus, have the potential for errors.

Glue programming is very efficient for automation of clinical tasks that often use several systems. However, caution should be used during implementation, because each component is independent, so the behavior of a component and its interactions can change during the execution of the script. In addition, various versions of the components may behave differently. Therefore, updates of one component may break the glue code, so a rigorous software QA program is necessary. We have not experienced any errors due to software updates in this specific TBI script, but this has happened in other scripts in clinical use. For example, when a major update to the TPS occurred (e.g., Aria v13.6 ‐> v15.6), a change to the ESAPI API script language required update to certain variables used in the script, and required recompiling of scripts.

## 5 CONCLUSIONS

In conclusion, while manual techniques may be well‐validated, they are time‐intensive and have large potential for error. Automation in the form of “Glue” programming reduces the barrier to fully automating treatment planning procedures, and shows great potential to improve clinical efficiency and safety within the treatment planning workflow for special procedures. This work shows both the value of incident learning systems (ILS) in practice knowledge dissemination, and shows the how automation of clinical processes and less reliance on human intervention has the potential for risk reduction.

## CONFLICT OF INTEREST

Dr. Thomas has nothing to disclose. No funding was received.
